# THE VULKAN TECHNIQUE: A NOVEL OSTOMY-CLOSURE TECHNIQUE THAT REDUCES COMPLICATIONS AND OPERATIVE TIMES

**DOI:** 10.1590/0102-6720201700020013

**Published:** 2017

**Authors:** Felix KRENZIEN, Christian BENZING, Fabian HARDERS, Tido JUNGHANS, Gyurdhan RASIM, Claudia BOTHE, Johann PRATSCHKE, Ricardo ZORRON

**Affiliations:** 1 Center of Innovative Surgery (ZIC), Department of Surgery, Campus Virchow Klinikum and Campus Mitte, Charité-Universitätsmedizin Berlin, Berlin, Germany;; 2 Berlin Institut of Health (BIH), Berlin, Germany;; 3 Department of General, Visceral, Thorax and Vascular Surgery, Clinic Bremerhaven Reinkenheide, Bremerhaven, Germany

**Keywords:** Vulkan technique, Ostomy, Ileostomy, Colostomy, Surgical site infection.

## Abstract

**Background:**

Ostomy reversals remain at high risk for surgical complications. Indeed, surgical-side infections due to bacterial contamination of the stoma lead to revision surgery and prolonged hospital stay.

**Aim:**

To describe the novel vulkan technique of ostomy reversal that aims to reduce operative times, surgical complications, and readmission rates.

**Methods:**

Ostomy closure was performed using the vulkan technique in all patients. This technique consists of external intestinal closure, circular skin incision and adhesiolysis, re-anastomosis, and closure of the subcutaneous tissue in three layers, while leaving a small secondary wound through which exudative fluid can be drained. The medical records of enterostomy patients were retrospectively reviewed from our hospital database.

**Results:**

The vulkan technique was successfully performed in 35 patients mainly by resident surgeons with <5 years of experience (n=22; 62.8%). The ileostomy and colostomy closure times were 53 min (interquartile range [IQR], 41-68 min; n=22) and 136 min (IQR: 88-188 min; n=13; p<0.001), respectively. The median hospital stay was seven days (IQR: 5−14.5 days); the length of hospital stay did not differ between ileostomy and colostomy groups. Major surgical complications occurred only in patients who underwent colostomy closure following the Hartmann procedure (n=2); grade≥IIIb according Clavien-Dindo classification.

**Conclusion:**

The vulkan technique was successfully applied in all patients with very low rates of surgical-site infections. Off note, residents with limited surgical experience mainly performed the procedure while operating time was less than one hour.

## RESUMO

**Racional:**

O procedimento de reversão de ileostomia ou colostomia após procedimento cirúrgico colônico permanecem com alto risco de complicações cirúrgicas. De fato, as infecções do sítio cirúrgico, devido à inerente contaminação bacteriana da operação, levam às operações de revisão e hospitalização prolongadas.

**Objetivo:**

O presente estudo visa descrever a técnica vulkan de reversão de ostomia, avaliando tempos operatórios, complicações cirúrgicas e taxas de readmissão.

**Métodos:**

O fechamento de ostomia foi realizado utilizando a técnica vulkan em todos os pacientes. Ela consiste em incisão cutânea circular, reanastomose, fechamento da aponeurose e fechamento do tecido subcutâneo em três camadas, deixando uma pequena ferida secundária através da qual se pode drenar o líquido exsudativo. A documentação dos pacientes com enterostomia foram revisadas retrospectivamente a partir da base de dados do hospital.

**Resultados:**

A técnica vulkan foi realizada com sucesso em 35 pacientes, principalmente por cirurgiões residentes com menos de cinco anos de experiência (n=22; 62,8%). Os tempos de ileostomia e fechamento da colostomia foram 53 min (41-68 min; n=22) e 136 min (88-188 min; n=13; p<0,001), respectivamente. A média da permanência hospitalar foi de sete dias (5-14,5 dias); o tempo de internação não diferiu entre os grupos de ileostomia e colostomia. As complicações cirúrgicas maiores ocorreram somente nos pacientes que se submeteram ao fechamento da colostomia após o procedimento de Hartmann (n=2, grau ≥IIIb de acordo com a classificação de Clavien-Dindo).

**Conclusão:**

A técnica vulkan foi aplicada com sucesso em todos os pacientes com taxas muito baixas de infecções no local cirúrgico. Além disso, as operações foram realizadas principalmente por residentes com experiência cirúrgica limitada, resultando em tempos operatórios inferiores a uma hora.

## INTRODUCTION

Stomata are artificial openings on the body surface that lead to a hollow organ. In gastrointestinal surgery, stomata are created to divert the flow of feces away from a certain site and out of the body. Stomata can be reversed after patients have recovered and the initial pathology has resolved. The benefits of stomata for different underlying diseases have been widely reported^14^ ^,^ ^17^. In contrast, the surgical procedure of stomata closure is less well investigated, and can be affected by minor and major complications^3^. Although stoma reversal is widely performed in clinical practice, it is associated with high complication rates, which affect patient outcomes and increase hospitalization costs. The development of fistulas, fascial dehiscence, or small bowel obstruction can necessitate revision surgery and significantly impact the outcomes of patients with gastrointestinal stomata. Furthermore, surgical-site infections (SSIs) have been reported to occur in up to 41% of patients with stomata^10^ ^,^ ^13^. 

To reduce stoma-related complications, various researchers have analyzed the different steps of the stomata reversal. Studies have been conducted to determine whether a hand-sewn anastomosis is superior to a stapled anastomosis. Based solely on randomized trials, stapledfunctional anastomoses are associated with fewer anastomotic leakages than hand-sewn anastomoses^2^. In addition, the conventional stoma-closure procedure has been performed laparoscopically. However, this is technically challenging, and is typically not performed by new or resident surgeons. Furthermore, owing to the complexity of the surgery, the procedure is prolonged, and has been associated with a mean operative time of up to 109 min^8^ ^,^ ^11^ ^,^ ^12^. Nevertheless, the laparoscopic procedure may be preferred in patients who require hernia repair and adhesiolysis, as it provides superior exposure of the surgical field and better access to the abdominal cavity. 

Fascial and skin closure following stoma reversal are crucial for minimizing complications and facilitating recovery. Drainage tubes for the evacuation of exudative and suppurative fluids,and even delayed primary skin closure have been used to reduce the incidence of SSIs after stoma reversal^8^. Conventional closure through a linear incision enlarges the skin defect. In 1997, Banerjee et al. described the purse-string closure technique of stoma reversal^1^. Since then, systematic reviews have shown that this procedure does reduce SSI rates, but its impact on the length of hospital stay is questionable^9^ ^,^ ^10^ ^,^ ^13^. Interestingly, readmission rates have not been reported in most studies on stoma closure. One retrospective analysis of 351 enterostomy reversals reported a readmission rate of 12.5%^7^; moreover, this rate was linked to long operative times, and increased intraoperative complications and hospital stay.

To overcome the above shortcomings in ostomy closure, we developed technique for ostomy closure termed the “vulkan” technique. In this technique, intestinal re-anastomosis is followed by the circular closure of the subcutaneous tissue in layers with the retention of a small secondary defect through which exudative and suppurative fluids can be drained. This scenario is likened to a volcano or “vulkan” in German, hence the name. In this study, we validated the vulkan technique in a clinical series of 35 patients with loop ileostomy and end colostomy, in terms of patient outcomes, operative time, and surgical complications.

## METHODS

### Study design

This retrospective study was conducted at the Department of General, Visceral, Thoracic and Vascular Surgery, Bremerhaven Hospital, Germany. Between July 2011 and April 2013, 35 consecutive patients with protective loop ileostomy or end colostomy due to different underlying diseases underwent ostomy closure by the vulkan technique. The end colostomy had been created as part of the Hartmann procedure. All patients provided informed consent prior to undergoing the vulkan procedure.

The procedure was mainly performed by resident surgeons with less than five years of surgical experience. The medical records of the patients were documented in our hospital database. Follow-up data and readmission rates were assessed as well as operative times and surgical complications according to the Clavien-Dindo classification^3^. A single dose of an antibiotic (500 mg metronidazole/2 g cefuroxime) was prophylactically administered before the surgery.

### The vulkan technique of stoma closure

**FIGURE 1:**
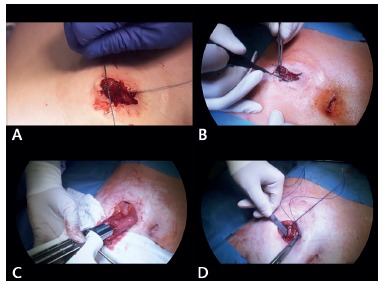
The vulkan technique for ileostomy closure, part 1: A) a running suture (Vicryl 0-0) is temporarily placed at the mucosa level (without touching the skin) to close the ostomy and avoid stool spillage during the procedure; B) a circular incision is made around the ostomy to mobilize the ileum and it is important to make the incision at the border between the mucosa and the skin to avoid skin resection and create as small a wound defect as possible; C) progressive adhesiolysis of the bowel is performed followed by a stapled latero-lateral linear anastomosis; D) the anastomosis can be oversewn with running polydioxanone (PDS) 4-0 sutures. Next, the abdominal fascia is closed with a full-thickness running suture (PDS 2-0).

Stoma closure was performed as follows: external closure of the intestinal mucosa to avoid fecal spillage (Figure 1A); circular incision around the ostomy to release adhesions and mobilize the bowel (Figure 1B); hand-sewn or stapled anastomosis (Figure 1C); and fascial closure with full-thickness running polydioxanone sutures (Figure 1D). Afterwards, sutures were placed in three consecutive layers, forming a triple crown. (Supplementary video, https://youtu.be/yw-N-egaBNA).

In contrast to the linear closure technique, drainage tubes were not placed after the vulkan procedure. Exudative and suppurative fluidswere evacuated through the small secondary defect remaining in the center of the wound, as all three circular sutures provide incomplete closure at each level.

### Statistical analysis

Statistical analysis was performed using SPSS version 20 (IBM Corporation, Armonk, NY, USA). Continuous variables were expressed as medians, and categorical values were expressed as percentages. If applicable, the interquartile range (IQR; 25−75%) was expressed. Differences between groups were analyzed using the Mann-Whitney U test with Bonferroni correction or the Student t-test as appropriate. P values smaller than 0.05 were considered significant.

## RESULTS

The baseline characteristics of the patients are shown in Table 1. The vulkan technique was successfully performed in all 35 patients, of whom 13 (37.1%) underwent colostomy reversal, and 22 (62.8%) underwent ileostomy reversal. Most patients with ileostomy had colorectal cancer (45.7%, n=16). All colostomies had been carried out in the course of a Hartmann procedure including a midline laparotomy followed by adhesiolysis. The vulkan procedure was performed mainly by resident surgeons (62.8%; n=22) with limited surgical experience (less than fiveyears).

**TABLE 1:** Baseline characteristics of the study population

Variable	Patients (n = 35)
Age (years)	59.75 (52-72)
Gender	
Male	19 (54.3%)
Female	16 (45.7%)
Length of stay	7 (5.2-12)
Colostomy	13 (37.1%)
Ileostomy	22 (62.8%)
Underlying disease	
Colorectal cancer	16 (45.7%)
Diverticulosis	6 (17.1%)
Other	9 (25.7

The procedure time was significantly shorter for ileostomy closure than for colostomy closure (p<0.001; Figure 3A). The mean procedure times for colostomy reversal and ileostomy reversal were 136 min (IQR: 88-188 min) and 53 min (IQR: 41-68 min), respectively. Major surgical complications occurred only in the patients who underwent colostomy closure (n=2; grade≥IIIb; Figure 3B). 

**FIGURE 2:**
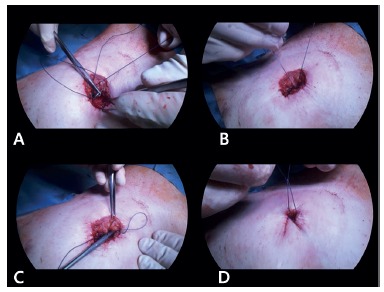
The vulkan technique for ileostomy closure, part 2: A and B) a circular pre-fascial subcutaneous suture is placed using Vicryl 1-0, and at this deep subcutaneous layer includes the fascial layer of the rectus abdominis to reduce the potential space for fluid accumulation; C) a second circular superficial subcutaneous suture is placed using Vicryl 1-0 as well; D) a third circular subcuticular suture is placed (Vicryl 1-0), forming a small wound opening. The three suture layers characterize the vulkan technique. Exudative fluid can be constantly evacuated from the remaining wound.

**FIGURE 3:**
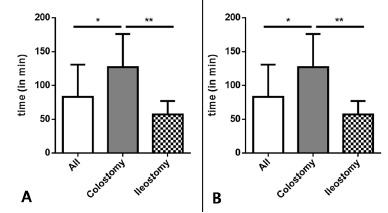
Operative characteristics: A) operative times for ostomy reversal by the vulkan technique; B) postoperative morbidity as determined according to the Clavien-Dindo classification. Major complications were defined as complications with grade≥IIIb.

Values are expressed as mean±SD; *p<0.05; **p<0.001; Mann-Whitney U-test was used to compare groups

One patient developed fascial dehiscence caused by wound infection, and another suffered ischemic colitis. Both were treated using revision surgery. SSIs occurred in three patients (8.5%; grade II), and were successfully managed with conventional dressings.

**FIGURE 4:**
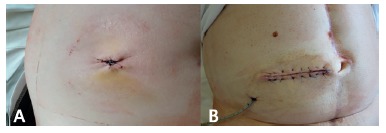
Vulkan technique vs. conventional ostomy closure: A) the vulkan technique resulted in a small, circular scar, and exudative fluids were constantly drained through the remaining wound defect; B) cosmetic results of conventional enterostomy closure. Conventional linear closure leads to large scars, and exudative wound fluids can only be evacuated by the placement of a drainage tube.

The cosmetic results were acceptable, and consisted of a small scar (Figure 4A) unlike the linear closure (Figure 4B). The wound shrunk in diameter, and the initial elevation of the tissue was decreased. The remaining wound opening was used to evacuate exudative fluids and thus prevent secondary infections. The mean length of stay following surgery was seven days (IQR: 5−14.5 days), and did not significantly differ between the ileostomy (6.5 days; IQR: 5-13.3 days) and colostomy groups (7.5 days; IQR: 5.5-15.5 days). None of the patients was readmitted to the hospital due to complications of stoma reversal.

## DISCUSSION

The novel vulkan technique was successfully applied for enterostomy reversal in this clinical series of 35 patients. The technique was found to be simple and feasible as indicated by the proportion of resident surgeons who were able to successfully perform the surgery despite their limited surgical experience (less than five years). Moreover, the overall procedure time was less than 1 h in the case of ileostomy reversal. The cosmetic results were acceptable, and continuous evacuation of potentially exudative and suppurative fluids was enabled through the small wound defect.

The vulkan technique is characterized by the placement of consecutive, circular subcutaneous and subcuticular sutures following re-anastomosis and fascial closure. Indeed, there are major differences between this technique and current stoma-closure techniques^9^. Conventional linear skin closure mostly involves a transverse linear incision. The skin is tightly adapted, and as fluid will remain enclosed underneath, the patient is prone to develop superinfection. Drainage can resolve the issue with fluid evacuation, but the incidence of SSIs after conventional linear closure remains high, up to 29.6%^10^. In addition to the high complication rate, conventional linear closure is associated with a larger scar and inferior cosmetic results (Figure 4B)^10^. Purse-string stoma closure, like the vulkan technique, is performed using a circular incision around the enterostomy. However, the two techniques are distinct^1^. The vulkan technique involves subcutaneous sutures placed in three consecutive layers (triple crown). Moreover, the first (deepest) layer of sutures includes the fascial layer of the rectus abdominis to reduce the potential space for fluid accumulation. This is especially important in obese patients with a certain amount of subcutaneous fat. When single-layer closure is performed, large wound defects and SSIs will occur in high-risk patients. Interestingly, Banerjee et al. first proposed this open technique for ostomy closure but using only a single purse-string suture^1^. In systematic reviews, this procedure was able to reduce SSI rates as compared to linear closure, but had no impact on length of stay or operative time^9^ ^,^ ^10^ ^,^ ^13^. 

The vulkan procedure was newly introduced to our department. Thus, there was a learning curve for the procedure. Nevertheless, the operative time was less than 1 h for ileostomy reversal. The short operative time might reflect the ease of this technique, and this might be significant in an economical context. Prolonged operative times are known to be highly linked to readmission rates (odds ratio, 1.6)^15^ and increased complication rates^15^. In contrast, short operative times do not necessarily reflect superior clinical outcomes; for instance low recurrence rate has been reported for hernia repair when operative time was prolonged (n=123,917)^16^. Taken together, the operative time for the vulkan technique solely indicates its feasibility and economic viability for ostomy closure in our specific clinical setting.

None of our patients required readmission after enterostomy closure. Although we cannot rule out the possibility that patients were admitted to other hospitals, most of the patients attended follow-up in our cancer program. Interestingly, readmission rates are rarely considered in clinical trials for stoma-closure techniques^7^. This bias profoundly affects the evaluation of closure techniques and the identification of all complications following the surgery. This issue seems to be a common problem in surgical and clinical trials. In contrast, cost control and quality management studies necessitate the assessment of readmission rates^4^. 

The anastomosis technique was not considered in our study and is not part of the vulkan technique. Whether a hand-sewn anastomosis is superior to stapled anastomosis remains uncertain. A Cochrane analysis of randomized trials reported that a stapledfunctional end-to-endileocolic anastomosis was associated with fewer complications than a hand-sewn anastomosis^2^. The anastomosis can also be created laparoscopically. The significance of laparoscopic reversal remains controversial, though selected patients may benefit from it^18^. 

The present study has certain limitations. First, we did not compare our technique with conventional linear closure or PSC (Partial Subcutaneous Closure). This might be the most important drawback, since solely the vulkan technique was used for enterostomy reversal in this study. Clearly, it would be very interesting for upcoming clinical trials to validate this technique by comparing it with PSC or conventional closure. Second, this was a retrospective analysis, and was inferior to prospective studies. Third, the study was underpowered, as the number of patients was small, and all patients were treated by the proposed technique. Fourth, the vulkan technique was used for both ileostomy and colostomy closure. This led to differences in procedure times and complication rates. Compared to ileostomy closure, closure of the colostomy following the Hartmann procedure was associated with higher rates of major complications and significantly longer operative times. This difference is attributable to the necessity of a laparotomy and consecutive adhesiolysis in the colostomy patients, and is consistent with the current literature^5^. 

## CONCLUSION

Preliminary results of the novel vulkan technique for ostomy reversal indicate that this technique is feasible, safe, and easy to use, and yields good cosmetic results. These findings were underscored by the fact that most operations were performed by young surgeons, and yet, the overall operative time was less than 1 h for ileostomy closure. Further clinical trials are required to evaluate the proposed technique and compare it with established closure techniques.

## Supplementary video:

https://youtu.be/yw-N-egaBNA
